# Interplay between 3′-UTR polymorphisms in the vascular endothelial growth factor (VEGF) gene and metabolic syndrome in determining the risk of colorectal cancer in Koreans

**DOI:** 10.1186/1471-2407-14-881

**Published:** 2014-11-25

**Authors:** Young Joo Jeon, Jong Woo Kim, Hye Mi Park, Hyo Geun Jang, Jung O Kim, Jisu Oh, So Young Chong, Sung Won Kwon, Eo Jin Kim, Doyeun Oh, Nam Keun Kim

**Affiliations:** Institute for Clinical Research, School of Medicine, CHA University, 351, Yatap-dong, Bundang-gu, Seongnam, 463-712 South Korea; Department of Biomedical Science, College of Life Science, CHA University, Seongnam, 463-712 South Korea; Department of Surgery, School of Medicine, CHA University, Seongnam, 463-712 South Korea; Department of Internal Medicine, School of Medicine, CHA University, 351, Yatap-dong Bundang-gu, Seongnam, 463-712 South Korea; Department of Medicine, College of Medicine, Chung-Ang University, Seoul, 456-756 South Korea

**Keywords:** *VEGF*, 3′-UTR, Polymorphism, Colorectal cancer, Metabolic syndrome

## Abstract

**Background:**

Polymorphisms in angiogenesis-related genes and metabolic syndrome (MetS) risk factors play important roles in cancer development. Moreover, recent studies have reported associations between a number of 3′-UTR polymorphisms and a variety of cancers. The aim of this study was to investigate the associations of three *VEGF* 3′-UTR polymorphisms (1451C > T [rs3025040], 1612G > A [rs10434], and 1725G > A [rs3025053]) and MetS with colorectal cancer (CRC) susceptibility in Koreans.

**Methods:**

A total of 850 participants (450 CRC patients and 400 controls) were enrolled in the study. The genotyping of *VEGF* polymorphisms was performed by TaqMan allelic discrimination assays. Cancer risks of genetic variations and gene-environment interactions were assessed by adjusted odds ratios (AORs) and 95% confidence intervals (CIs) of multivariate logistic regression analyses.

**Results:**

*VEGF* 1451C > T was significantly associated with rectal cancer risk (Dominant model; AOR =1.58; 95% CI = 1.09 - 2.28; *p* = 0.015) whereas *VEGF* 1725G > A correlated with MetS risk (Dominant model; AOR =1.61; 95% CI =1.06 - 2.46; *p* = 0.026). Of the gene-environment combined effects, the interaction of *VEGF* 1451C > T and MetS contributed to increased rectal cancer risk (AOR = 3.15; 95% CI = 1.74 - 5.70; *p* < .001) whereas the combination of *VEGF* 1725G > A and MetS was involved with elevated colon cancer risk (AOR = 2.68; 95% CI = 1.30 - 1.55; *p* =0.008).

**Conclusions:**

Our results implicate that *VEGF* 1451C > T and 1725G > A may predispose to CRC susceptibility and the genetic contributions may be varied with the presence of MetS.

**Electronic supplementary material:**

The online version of this article (doi:10.1186/1471-2407-14-881) contains supplementary material, which is available to authorized users.

## Background

Colorectal cancer (CRC) is the third most common type of cancer and the second leading cause of cancer-related mortality in Western countries [1]. The prognosis of patients with CRC depends on the tumor stage at the time of diagnosis. However, over 57% of patients have regional or distant spread of tumor cells at the time of diagnosis [2]. The pathogenesis of CRC usually follows a stepwise progression from benign polyp to invasive adenocarcinoma. In colorectal carcinogenesis, the unique molecular and genetic changes that occur within cells result in a specific CRC phenotype. This phenotype is associated with variable tumor behaviors that are relevant to the prognosis and the response to specific therapies. As a result, the term “CRC” no longer refers to a single disease, but rather a heterogeneous group of diseases caused by differential genetic/epigenetic backgrounds. In this respect, many ongoing studies are aimed at assessing biomarkers as potential predictors of prognosis or response to therapy, which will most likely lead to the individualized management of the disease.

Tumor angiogenesis is important to tumor growth, as evidenced by results showing that tumor growth is dependent on angiogenesis. Furthermore, tumors are able to produce diffusible angiogenic molecules [3], including vascular endothelial growth factor (VEGF). VEGF is a key regulator of angiogenesis with several functions that serve to enhance tumor progression. These functions include enhancing vascular permeability, inducing endothelial cell migration and division, inducing serine protease activity, inhibiting either the apoptosis of endothelial cells or maturation of dendritic cells, and inducing angiogenesis [4, 5]. The human *VEGF* gene is located on chromosome 6 and organized into eight exons and seven introns, which encode several different isoforms due to alternative splicing. There are five well-studied *VEGF* polymorphisms that have been linked to CRC: -2578C > A, -1498T > C, -1154G > A, -634G > C, and 936C > T. However, their genetic associations with CRC have been inconsistent [6]. Recent papers have shown some clinical impacts of polymorphisms in the 3′-UTR of certain genes, which may potentially bind to specific miRNAs in various cancers [7–10]. However, variants in the *VEGF* 3′-UTR have not been studied.

The metabolic syndrome (MetS) is a portfolio of metabolic disorders, including abdominal obesity, increased blood pressure (BP), abnormal glucose metabolism, and dyslipidemia [11]. Previous reports show that components of MetS are associated with CRC susceptibility [12, 13]. A previous study found a 35% increased CRC risk associated with high BP [14] and there was a similar finding in another prospective epidemiologic study [15]. Adult-onset diabetes mellitus (DM) has generally been associated with a higher risk of CRC [16–20] and elevated blood glucose levels correlated with a significantly elevated CRC risk (Relative risk [RR] = 1.80) [21]. However, gene-environment combined effects of MetS for CRC susceptibility have been infrequently found in previous published database.

In the *VEGF* gene, there are four known 3′-UTR polymorphisms (936C > T [rs3025039], 1451C > T [rs3025040], 1612G > A [rs10434], 1725G > A [rs3025053]). The *VEGF* 1451TT genotype presented a significant log-rank *p* value in non-small-cell lung cancer survival [22]. The *VEGF* 1612A allele was associated with increased gastric cancer risk [23]. The 936C > T polymorphism and its link to CRC susceptibility have been published by our laboratory and others [6, 24]. The other three single nucleotide polymorphisms (SNPs) in the *VEGF* 3′-UTR, 1451C > T, 1612G > A, and 1725G > A, are poorly understood in the context of their genetic contributions to CRC susceptibility. The purpose of this study was to investigate whether these polymorphisms of *VEGF* 3′-UTR correlate with CRC susceptibility and the genetic contributions are modified by the presence of MetS.

## Methods

### Study population

We conducted a case–control study of 850 individuals. Four hundred and fifty patients diagnosed with CRC at CHA Bundang Medical Center (Seongnam, South Korea) were enrolled from June 2004 to January 2009. This study only included CRC patients who had undergone surgical resection with a curative intent and who had histologically confirmed adenocarcinoma. Within the CRC cohort, 264 consecutive patients with colon cancer and 186 consecutive patients with rectal cancer underwent primary surgery. Tumor staging of CRCs was performed according to the sixth edition of the American Joint Committee on Cancer (AJCC) staging manual. The control group consisted of 400 individuals randomly selected following a health screening. This screening excluded patients with a history of cancer. Individuals were diagnosed with MetS if they possessed three or more of the following five risk factors: body mass index (BMI) ≥25.0 kg/m^2^; triglycerides (TG) ≥150 mg/dL; high density lipoprotein-cholesterol (HDL-C) <40 mg/dL for men or <50 mg/dL for women; BP ≥130/85 mmHg or currently taking anti-hypertension medication; and fasting blood sugar (FBS) ≥100 mg/dL or currently taking hypoglycemic medication. All study subjects were ethnic Koreans and provided written informed consent. The study protocol was approved by the Institutional Review Board of CHA Bundang Medical Center, Seongnam, South Korea.

### Phenotype measurements

Anthropometric measurements included BMI. Systolic and diastolic BP of subjects were measured in the seated position after 10 min of rest. For measurements of physiological parameters, 3 ml blood was obtained after fasting overnight. The hexokinase method was employed to measure FBS levels; samples were analyzed in duplicate by an automated analyzer (TBA 200FR NEO, Toshiba Medical Systems, Tokyo, Japan). TG and HDL-C levels were determined by enzymatic colorimetric methods using commercial reagent sets (TBA 200FR NEO, Toshiba Medical Systems).

### Genotyping

DNA was extracted from leukocytes using a G-DEX™ II Genomic DNA Extraction kit (Intron Biotechnology, Seongnam, Korea) according to the manufacturer’s instructions. *VEGF* genotypes were analyzed by TaqMan allelic discrimination analysis. Genotyping of the *VEGF* 1451C > T, *VEGF* 1612G > A, and *VEGF* 1725G > A polymorphisms was determined using real-time polymerase chain reaction (PCR) (RG-6000, Corbett Research, Australia) for allelic discrimination. Primers and TaqMan probes were designed using Primer Express Software (version 2.0) and synthesized and supplied by Applied Biosystems (Foster City, CA, USA). The reporter dyes used were FAM and JOE. The primer sequences for amplification are as follows: *VEGF* 1451C > T: forward 5′- ACG GAC AGA AAG ACA GAT CAC AG -3′ and reverse 5′- CCC AAA GCA CAG CAA TGT C -3′. The selected probes were 5′-FAM- TGA GGA CAC CGG CTC TGA CC -TAMRA-3′ (C allele detecting probe) and 5′-JOE- TGA GGA CAC TGG CTC TGA CC -TAMRA-3′ (T allele detecting probe). *VEGF* 1612G > A: forward 5′- TTC GCT TAC TCT CAC CTG CTT C -3′ and reverse 5′- GCT GTC ATG GGC TGC TTC T -3′. The selected probes were 5′-FAM- CCC AGG AGG CCA CTG GCA -TAMRA-3′ (G allele detecting probe) and 5′-JOE- CCC AGG AGA CCA CTG GCA -TAMRA-3′ (A allele detecting probe). *VEGF* 1725G > A: forward 5′- CAT GAC AGC TCC CCT TCC T -3′ and reverse 5′- TGG TTT CAA TGG TGT GAG GAC -3′. The selected probes were 5′-FAM- CTT CCT GGG GTG CAG CCT AA -TAMRA-3′ (G allele detecting probe) and 5′-JOE- CTT CCT GGG ATG CAG CCT AA -TAMRA-3′ (A allele detecting probe). For each polymorphism, 30% of the PCR assays were randomly selected and repeated, followed by DNA sequencing, to validate the experimental findings. Sequencing was performed using an ABI 3730xl DNA Analyzer (Applied Biosystems, Foster City, CA, USA). The concordance of the quality control samples was 100%.

### Quantitative real-time PCR

To perform quantitative real-time PCR (qRT-PCR), total RNA was extracted from 47 tumor and tumor-adjacent tissues from 47 CRC patients by using TRIzol Reagent (Invitrogen, Grand Island, NY, USA) according to the manufacturer’s instructions. cDNA was made from total RNA with the SuperScript III First-Strand Synthesis System (Invitrogen, Grand Island, NY, USA). Measurement of the VEGF mRNA was determined using real-time PCR (RG-6000, Corbett Research, Australia). The expression level of VEGF mRNA in 47 tumor and tumor-adjacent tissues was compared by a comparative C_T_ (2^-ΔΔC^_T_) method with housekeeping internal control, 18 s rRNA. The primer sequences for amplification are as follows: 18 s rRNA: forward 5′- AAC TTT CGA TGG TAG TCG CCG -3′ and reverse 5′- CCT TGG ATG TGG TAG CCG TTT -3′. VEGF: forward 5′- TGA GCT TCC TAC AGC ACA AC -3′ and reverse 5′- ATT TAC ACG TCT GCG GAT CTT -3′.

### Statistical analysis

To analyze baseline characteristics, odds ratios (ORs) and 95% confidence intervals (95% CIs) from univariate logistic regression were used to compare patient and control baseline data. Genetic associations of *VEGF* 1451C > T, 1612G > A, and 1725G > A polymorphisms with MetS and CRC susceptibility were calculated using adjusted odds ratios (AORs) and 95% CIs from multivariate logistic regression. The variables age, gender, and MetS risk factors were selected as adjustment variables. To estimate MetS and CRC risk, we used three genetic susceptibility models: additive, dominant, and recessive. All *VEGF* 3′-UTR genotypes were converted into numeric values for logistic regression according to their genotypes. Wild homozygotes were assigned “0” in all models. Heterozygotes were assigned “1” in additive and dominant models and “0” in the recessive model. Mutant homozygotes were assigned “1” in dominant and recessive models and “2” in the additive model. Gene-environment interaction analysis was performed using the open-source multifactor dimensionality reduction (MDR) software package (v.2.0) available from http://www.epistasis.org. The comparisons of relative VEGF mRNA expression were analyzed by Mann–Whitney, Krusukal-Wallis, and Wilcoxon signed rank tests. Analyses were performed using GraphPad Prism 4.0 (GraphPad Software Inc., San Diego, CA, USA) and Medcalc version 12.7.1.0 (Medcalc Software, Mariakerke, Belgium). Haplotypes for multiple loci were estimated using the expectation-maximization algorithm with SNPAlyze (Version 5.1; DYNACOM Co, Ltd, Yokohama, Japan).

## Results

In this study, we collected data for 450 CRC patients (264 colon cancer [CC] and 186 rectal cancer [RC] patients), including 212 men and 238 women. Both CC and RC groups have higher portion of tumor size ≥5 cm and tumor node metastasis (TNM) stage II/III (Table 1). The presence of MetS was associated with CRC susceptibility (CC group: OR = 1.90; 95% CI = 1.35 - 2.66; *p* < .001; RC group: OR = 2.07; 95% CI = 1.43 - 3.01; *p* < .001). Of MetS risk factors, lower HDL-C (<40 mg/dL for men or <50 mg/dL for women) strongly contributed to CC and RC risk (CC group: OR = 3.09; 95% CI = 2.18 - 4.37; *p* < .001; RC group: OR = 3.40; 95% CI = 2.32 - 4.97; *p* < .001). Table 2 shows the distributions of genotypes and haplotypes for *VEGF* 1451C > T, 1612G > A, and 1725G > A polymorphisms stratified by the presence of MetS. The *VEGF* 3′-UTR genotype frequencies of controls were consistent with the Hardy-Weinberg equilibrium. Table 3 presents AOR values for MetS, CC, and RC risk by *VEGF* 3′-UTR polymorphisms. *VEGF* 1451C > T was significantly associated with RC risk (Dominant model: AOR = 1.58; 95% CI = 1.09 - 2.28; *p* = 0.015) whereas *VEGF* 1725G > A correlated with MetS risk (Dominant model: AOR = 1.61; 95% CI = 1.06 - 2.46; *p* = 0.026). As a similar pattern, in haplotype analysis, *VEGF* 1451T/1612G/1725G contributed to RC risk (AOR = 1.40; 95% CI = 1.03 - 1.92; *p* = 0.030) while *VEGF* 1451C/1612A/1725A was involved with MetS risk (AOR = 1.54; 95% CI = 1.02 - 2.34; *p* = 0.041).

**Table 1:** **Baseline characteristics in colorectal cancer patients and control subjects**

Characteristics	Control	CC	OR (95% CI)	***p***	RC	OR (95% CI)	***p***
N	400	264			186		
Age (mean±SD)	60.89 ± 11.72	61.85 ± 12.85	1.01 (0.99 - 1.02)	0.320	62.33 ± 11.46	1.01 (1.00 - 1.03)	0.165
Gender (male), n (%)	170 (42.5)	124 (47.0)	1.20 (0.88 - 1.64)	0.257	88 (47.3)	1.21 (0.86 - 1.72)	0.275
Metabolic syndrome, n (%)	95 (23.8)	98 (37.1)	1.90 (1.35 - 2.66)	<.001	73 (39.2)	2.07 (1.43 - 3.01)	<.001
Anti-HTN drug or BP ≥ 130/85 mmHg, n (%)	157 (39.3)	159 (60.2)	2.34 (1.71 - 3.22)	<.001	120 (64.5)	2.81 (1.96 - 4.04)	<.001
Anti-DM drug or FBS ≥ 100 mg/dl, n (%)	166 (41.5)	149 (56.4)	1.83 (1.33 - 2.50)	<.001	104 (55.9)	1.79 (1.26 - 2.54)	0.001
TG ≥ 150 mg/dl, n (%)	135 (33.8)	67 (25.4)	0.67 (0.47 - 0.94)	0.022	46 (24.7)	0.65 (0.44 - 0.95)	0.029
BMI ≥ 25 kg/m^2^, n (%)	93 (23.3)	65 (24.6)	1.08 (0.75 - 1.55)	0.685	51 (27.4)	1.25 (0.84 - 1.85)	0.276
HDL-C < 40(male)/50(female) mg/dl, n (%)	78 (19.5)	113 (42.8)	3.09 (2.18 - 4.37)	<.001	84 (45.2)	3.40 (2.32 - 4.97)	<.001
Tumor size							
<5 cm	-	94 (35.6)	-	-	87 (46.8)	-	-
≥5 cm	-	170 (64.4)	-	-	99 (53.2)	-	-
TNM stage, n (%)							
I	-	22 (8.3)	-	-	20 (10.8)	-	-
II	-	118 (44.7)	-	-	71 (38.2)	-	-
III	-	94 (35.6)	-	-	79 (42.5)	-	-
IV	-	30 (11.4)	-	-	16 (8.6)	-	-

Colon cancer (CC), Rectal cancer (RC), Odds ratio (OR), Confidence interval (CI), Standard deviation (SD), Blood pressure (BP), Fasting blood sugar (FBS), Hypertension (HTN), Diabetes mellitus (DM), Triglycerides (TG), Body mass index (BMI), High density lipoprotein-cholesterol (HDL-C), Tumor node metastasis (TNM). ORs and *p* values were calculated by univariate logistic regression.

**Table 2:** **Genotype and haplotype frequencies of** ***VEGF*** **3′-UTR polymorphisms**

	Without MetS			With MetS		
Genotype/Haplotype	Control (n = 305)	CC (n = 166)	RC (n = 113)	Control (n = 95)	CC (n = 98)	RC (n = 73)
*VEGF* 1451CC	209 (68.5)	108 (65.1)	64 (56.6)	65 (68.4)	72 (73.5)	45 (61.6)
*VEGF* 1451CT	81 (26.6)	50 (30.1)	43 (38.1)	29 (30.5)	23 (23.5)	27 (37.0)
*VEGF* 1451TT	15 (4.9)	8 (4.8)	6 (5.3)	1 (1.1)	3 (3.1)	1 (1.4)
HWE*-p*	0.059	0.483	0.724	0.251	0.493	0.167
*VEGF* 1612GG	213 (69.8)	127 (76.5)	87 (77.0)	67 (70.5)	68 (69.4)	54 (74.0)
*VEGF* 1612GA	83 (27.2)	36 (21.7)	21 (18.6)	27 (28.4)	29 (29.6)	18 (24.7)
*VEGF* 1612AA	9 (3.0)	3 (1.8)	5 (4.4)	1 (1.1)	1 (1.0)	1 (1.4)
HWE*-p*	0.791	0.809	0.022	0.336	0.271	0.714
*VEGF* 1725GG	278 (91.1)	147 (88.6)	96 (85.0)	81 (85.3)	79 (80.6)	61 (83.6)
*VEGF* 1725GA	27 (8.9)	19 (11.4)	17 (15.0)	14 (14.7)	19 (19.4)	12 (16.4)
*VEGF* 1725AA	0 (0.0)	0 (0.0)	0 (0.0)	0 (0.0)	0 (0.0)	0 (0.0)
HWE*-p*	0.419	0.434	0.387	0.438	0.288	0.444
*VEGF* 1451C/1612G/1725G	398 (65.2)	221 (65.6)	140 (61.9)	130 (68.4)	134 (68.4)	96 (65.8)
*VEGF* 1451T/1612G/1725G	111 (18.2)	66 (19.9)	55 (24.3)	31 (16.3)	29 (14.8)	29 (19.9)
*VEGF* 1451C/1612A/1725G	74 (12.1)	26 (7.8)	14 (6.2)	15 (7.9)	14 (7.1)	9 (6.2)
*VEGF* 1451C/1612A/1725A	27 (4.4)	16 (4.8)	17 (7.5)	14 (7.4)	17 (8.7)	11 (7.5)
*VEGF* 1451C/1612G/1725A	0 (0.0)	3 (0.9)	0 (0.0)	0 (0.0)	2 (1.0)	1 (0.7)

Vascular endothelial growth factor (VEGF), Colon cancer (CC), Rectal cancer (RC), Metabolic syndrome (MetS), Hardy-Weinberg equilibrium (HWE).

**Table 3:** **Adjusted odds ratios for metabolic syndrome, colon cancer, and rectal cancer risks according to** ***VEGF*** **3′-UTR variants**

			MetS risk		CC risk		RC risk	
Genotype/Haplotype	Model	Wild type	AOR (95% CI)*	***p***	AOR (95% CI)**	***p***	AOR (95% CI)**	***p***
*VEGF* 1451C > T	Additive	CC	0.80 (0.61 - 1.05)	0.105	1.05 (0.79 - 1.39)	0.742	1.40 (1.03 - 1.91)	0.031
	Dominant	CC	0.85 (0.62 - 1.16)	0.295	1.04 (0.74 - 1.46)	0.813	1.58 (1.09 - 2.28)	0.015
	Recessive	CC	0.36 (0.14 - 0.96)	0.041	1.16 (0.53 - 2.57)	0.709	1.12 (0.45 - 2.80)	0.804
*VEGF* 1612G > A	Additive	GG	1.02 (0.76 - 1.36)	0.920	0.82 (0.59 - 1.12)	0.216	0.83 (0.58 - 1.18)	0.304
	Dominant	GG	1.11 (0.80 - 1.54)	0.521	0.81 (0.57 - 1.15)	0.239	0.74 (0.50 - 1.11)	0.149
	Recessive	GG	0.38 (0.11 - 1.33)	0.130	0.68 (0.21 - 2.21)	0.521	1.48 (0.52 - 4.19)	0.458
*VEGF* 1725G > A	Additive	GG	1.61 (1.06 - 2.46)	0.026	1.34 (0.83 - 2.16)	0.235	1.51 (0.90 - 2.55)	0.118
	Dominant	GG	1.61 (1.06 - 2.46)	0.026	1.34 (0.83 - 2.16)	0.235	1.51 (0.90 - 2.55)	0.118
	Recessive	GG	-	-	-	-	-	-
*VEGF* 1451C/1612G/1725G		Others	1.14 (0.92 - 1.43)	0.232	1.04 (0.82 - 1.31)	0.762	0.87 (0.67 - 1.12)	0.279
*VEGF* 1451T/1612G/1725G		Others	0.80 (0.61 - 1.05)	0.101	1.05 (0.79 - 1.41)	0.735	1.40 (1.03 - 1.92)	0.030
*VEGF* 1451C/1612A/1725G		Others	0.73 (0.50 - 1.07)	0.111	0.69 (0.47 - 1.02)	0.066	0.56 (0.34 - 0.90)	0.017
*VEGF* 1451C/1612A/1725A		Others	1.54 (1.02 - 2.34)	0.041	1.12 (0.69 - 1.80)	0.652	1.42 (0.86 - 2.36)	0.170

Adjusted odds ratio (AOR), Confidence interval (CI), Metabolic syndrome (MetS), Colon cancer (CC), Rectal cancer (RC), Vascular endothelial growth factor (VEGF). *AORs and *p* values were adjusted by age and gender. **AORs and *p* values were adjusted by age, gender, and MetS risk factors.

Because cancer risk is determined by a complex interplay of genetic and environmental factors, we calculated gene-environment combined effects between MetS and *VEGF* 3′-UTR polymorphisms. Preferentially, we sought to determine whether the contribution of *VEGF* 3′-UTR genetic variants to CRC susceptibility varied with the presence of MetS (Table 4). The significant involvements of *VEGF* 1451C > T (Dominant model: AOR = 1.67; 95% CI = 1.07 - 2.61; *p* = 0.023) and *VEGF* 1451T/1612G/1725G (AOR = 1.45; 95% CI = 1.00 - 2.09; *p* = 0.047) with RC risk were found in the absence of MetS. Table 5 displays gene-environment interaction effects between *VEGF* 3′-UTR polymorphisms and MetS. We examined all possible combinations of gene-environment interactions using MDR methods. The combination of *VEGF* 1451C > T and MetS was the best model to evaluate RC risk (Cross validation consistency = 9/10), while the combination of *VEGF* 1725G > A and MetS was the most appropriate model to assess CC risk (Cross validation consistency = 7/10). The interaction of *VEGF* 1451C > T and MetS contributed to increased RC risk (AOR = 3.15; 95% CI = 1.74 - 5.70; *p* < .001) whereas the combination of *VEGF* 1725G > A and MetS was involved with elevated CC risk (AOR = 2.68; 95% CI =1.30 - 1.55; *p* = 0.008).

**Table 4:** **Stratified effects of metabolic syndrome on colon cancer and rectal cancer risks by** ***VEGF*** **3′-UTR variants**

				CC risk		RC risk	
Genotype/Haplotype	Model	Subgroup	Wild type	AOR (95% CI)	***p***	AOR (95% CI)	***p***
*VEGF* 1451C > T	Additive	Without MetS	CC	1.12 (0.80 - 1.54)	0.513	1.42 (0.99 - 2.03)	0.055
		With MetS	CC	0.89 (0.50 - 1.59)	0.701	1.44 (0.77 - 2.67)	0.255
	Dominant	Without MetS	CC	1.18 (0.79 - 1.77)	0.416	1.67 (1.07 - 2.61)	0.023
		With MetS	CC	0.78 (0.41 - 1.49)	0.456	1.44 (0.74 - 2.78)	0.280
	Recessive	Without MetS	CC	0.99 (0.41 - 2.40)	0.985	1.09 (0.41 - 2.87)	0.868
		With MetS	CC	3.07 (0.30 - 31.00)	0.342	2.14 (0.13 - 35.71)	0.595
*VEGF* 1612G > A	Additive	Without MetS	GG	0.73 (0.49 - 1.07)	0.105	0.81 (0.53 - 1.24)	0.328
		With MetS	GG	1.10 (0.61 - 2.02)	0.746	0.97 (0.50 - 1.87)	0.928
	Dominant	Without MetS	GG	0.71 (0.46 - 1.09)	0.117	0.69 (0.42 - 1.14)	0.148
		With MetS	GG	1.13 (0.60 - 2.14)	0.704	0.93 (0.46 - 1.89)	0.850
	Recessive	Without MetS	GG	0.58 (0.15 - 2.17)	0.416	1.51 (0.49 - 4.61)	0.469
		With MetS	GG	0.78 (0.05 - 13.40)	0.867	1.72 (0.10 - 28.53)	0.705
*VEGF* 1725G > A	Additive	Without MetS	GG	1.34 (0.72 - 2.49)	0.360	1.82 (0.95 - 3.48)	0.072
		With MetS	GG	1.37 (0.62 - 2.98)	0.435	1.20 (0.51 - 2.85)	0.672
	Dominant	Without MetS	GG	1.34 (0.72 - 2.49)	0.360	1.82 (0.95 - 3.48)	0.072
		With MetS	GG	1.37 (0.62 - 2.98)	0.435	1.20 (0.51 - 2.85)	0.672
	Recessive	Without MetS	GG	-	-	-	-
		With MetS	GG	-	-	-	-
*VEGF* 1451C/1612G/1725G		Without MetS	Others	1.06 (0.80 - 1.40)	0.697	0.87 (0.63 - 1.19)	0.375
		With MetS	Others	0.95 (0.61 - 1.49)	0.840	0.80 (0.50 - 1.28)	0.354
*VEGF* 1451T/1612G/1725G		Without MetS	Others	1.13 (0.80 - 1.58)	0.495	1.45 (1.00 - 2.09)	0.047
		With MetS	Others	0.90 (0.51 - 1.58)	0.705	1.36 (0.77 - 2.42)	0.292
*VEGF* 1451C/1612A/1725G		Without MetS	Others	0.61 (0.38 - 0.97)	0.038	0.48 (0.26 - 0.86)	0.015
		With MetS	Others	1.04 (0.48 - 2.25)	0.930	0.87 (0.37 - 2.08)	0.760
*VEGF* 1451C/1612A/1725A		Without MetS	Others	1.09 (0.58 - 2.05)	0.795	1.75 (0.93 - 3.28)	0.081
		With MetS	Others	1.13 (0.53 - 2.43)	0.745	1.08 (0.47 - 2.51)	0.854

Adjusted odds ratio (AOR), Confidence interval (CI), Metabolic syndrome (MetS), Colon cancer (CC), Rectal cancer (RC), Vascular endothelial growth factor (VEGF). AORs and *p* values were adjusted by age and gender.

**Table 5:** **Combined effects of metabolic syndrome and** ***VEGF*** **3′-UTR variants on colon cancer and rectal cancer risks**

	CC risk				RC risk			
	Without MetS		With MetS		Without MetS		With MetS	
Genotype	AOR (95% CI)	***p***	AOR (95% CI)	***p***	AOR (95% CI)	***p***	AOR (95% CI)	***p***
*VEGF* 1451CC	1.00 (reference)		2.17 (1.43 - 3.28)	<.001	1.00 (reference)		2.22 (1.38 - 3.57)	0.001
*VEGF* 1451CT+TT	1.18 (0.79 - 1.77)	0.416	1.76 (0.98 - 3.16)	0.060	1.67 (1.07 - 2.61)	0.023	3.15 (1.74 - 5.70)	<.001
*VEGF* 1612GG	1.00 (reference)		1.68 (1.12 - 2.53)	0.013	1.00 (reference)		1.94 (1.24 - 3.01)	0.003
*VEGF* 1612GA+AA	0.71 (0.46 - 1.09)	0.117	1.81 (1.03 - 3.19)	0.040	0.69 (0.42 - 1.14)	0.148	1.66 (0.88 - 3.15)	0.117
*VEGF* 1725GG	1.00 (reference)		1.88 (1.29 - 2.73)	0.001	1.00 (reference)		2.17 (1.44 - 3.27)	<.001
*VEGF* 1725GA+AA	1.34 (0.72 - 2.49)	0.360	2.68 (1.30 - 5.55)	0.008	1.82 (0.95 - 3.48)	0.072	2.49 (1.11 - 5.57)	0.027

Adjusted odds ratio (AOR), Confidence interval (CI), Metabolic syndrome (MetS), Colon cancer (CC), Rectal cancer (RC), Vascular endothelial growth factor (VEGF). AORs and *p* values were adjusted by age and gender.

Finally, we quantified expression of VEGF in tissue samples and looked for differences in expression based on the tested haplotypes and genotypes. VEGF expression as a function of each *VEGF* 3′-UTR genotype or haplotype is presented in Table 6. Relative VEGF mRNA levels in samples with the 1451T/1612G/1725G were significantly decreased relative to the 1451C/1612G/1725G (*p <* .05). In contrast, relative VEGF mRNA levels in samples with the 1451C/1612A/1725A were significantly increased from levels in samples with the 1451C/1612G/1725G (*p <* .05). Table 7 shows VEGF expression between tumor and tumor-adjacent tissues according to studied polymorphisms. Relative VEGF mRNA expression of tumor-tissues is significantly increased in each wild genotype while not in each variant genotype. (Additional file 1: Table S1) displays the frequencies of MetS and *VEGF* 3′-UTR genotypes according to clinicopathological features of CRC. The frequency of *VEGF* 1451C > T was different between the CC and RC groups, but this difference was not statistically significant (*p =* 0.081).

**Table 6:** ***VEGF*** **mRNA expression levels (mean ± SE) according to** ***VEGF*** **3′-UTR genotypes and haplotypes**

	Tumor-adjacent (n = 47)	***p***	Tumor (n = 47)	***p***
*VEGF* 1451CC (n = 32)	1.00 ± 0.45	0.445	1.00 ± 0.47	0.158
*VEGF* 1451CT+TT (n = 15)	2.54 ± 2.13		0.02 ± 0.01	
*VEGF* 1612GG (n = 33)	1.00 ± 0.54	0.008	1.00 ± 0.67	0.156
*VEGF* 1612GA+AA (n = 14)	6.76 ± 4.24		3.67 ± 2.38	
*VEGF* 1725GG (n = 41)	1.00 ± 0.40	0.038	1.00 ± 0.58	0.011
*VEGF* 1725GA+AA (n = 6)	5.98 ± 4.30		7.61 ± 5.43	
*VEGF* 1451C/1612G/1725G (n = 61)	1.00 ± 0.35	0.064	1.00 ± 0.39	0.027
*VEGF* 1451T/1612G/1725G (n = 18)	2.61 ± 2.19		0.02 ± 0.01	
*VEGF* 1451C/1612A/1725G (n = 9)	2.72 ± 1.52		0.44 ± 0.42	
*VEGF* 1451C/1612A/1725A (n = 5)	7.92 ± 6.08		4.59 ± 3.28	

Standard error (SE), Vascular endothelial growth factor (VEGF). *p* values were calculated by Mann–Whitney and Kruskal-Wallis tests.

**Table 7:** ***VEGF*** **mRNA expression (mean ± SE) between tumor and tumor-adjacent tissues according to** ***VEGF*** **3′-UTR genotypes and haplotypes**

	Tumor-adjacent (n = 47)	Tumor (n = 47)	***p***
*VEGF* 1451CC (n = 32)	1.00 ± 0.45	75.37 ± 35.42	0.029
*VEGF* 1451CT+TT (n = 15)	1.00 ± 0.84	0.59 ± 0.30	0.125
*VEGF* 1612GG (n = 33)	1.00 ± 0.54	52.24 ± 35.00	0.001
*VEGF* 1612GA+AA (n = 14)	1.00 ± 0.63	28.36 ± 18.39	0.143
*VEGF* 1725GG (n = 41)	1.00 ± 0.40	30.65 ± 17.78	0.011
*VEGF* 1725GA+AA (n = 6)	1.00 ± 0.72	39.00 ± 27.83	0.203
*VEGF* 1451C/1612G/1725G (n = 61)	1.00 ± 0.35	67.56 ± 26.35	<.001
*VEGF* 1451T/1612G/1725G (n = 18)	1.00 ± 0.81	0.50 ± 0.25	0.133
*VEGF* 1451C/1612A/1725G (n = 9)	1.00 ± 0.56	10.93 ± 10.43	0.481
*VEGF* 1451C/1612A/1725A (n = 5)	1.00 ± 0.77	39.15 ± 27.98	0.210

Standard error (SE), Vascular endothelial growth factor (VEGF). *p* values were calculated by Wilcoxon signed rank test.

## Discussion

In the present study, we investigated whether *VEGF* 1451C > T, 1612G > A, and 1725G > A are involved with CRC susceptibility. We identified that *VEGF* 1451C > T was significantly associated with RC risk whereas *VEGF* 1725G > A correlated with MetS risk. Of the gene-environment combined effects, the interaction of *VEGF* 1451C > T and MetS contributed to increased RC risk whereas the combination of *VEGF* 1725G > A and MetS was involved with elevated CC risk. Furthermore, quantitative real-time PCR analysis revealed that relative VEGF mRNA expression in tumor tissues varied with *VEGF* 1451T/1612G/1725G and 1451C/1612A/1725A haplotypes. To our knowledge, *VEGF* 1451C > T and 1725G > A may play roles in CRC susceptibility.

Polymorphisms within the *VEGF* gene are a current topic of interest within the cancer epidemiology field. There are several association studies showing that *VEGF* -2578C > A, -1498T > C (-460T > C), -1154G > A, -634G > C (405G > C), and 936C > T correlate with CRC risk [6], and the following alleles: *VEGF* -2578A, -1498T, -1154A, -634C, and 936T: are associated with reduced VEGF expression [25–27]. Increased CRC incidence seems to occur in genotypes that cause both low (*VEGF* -2578A and 936T) and high (*VEGF* -1498C and -634G) VEGF expression [24, 28, 29].

Angiogenesis under physiological conditions is a strictly regulated process on many levels, including spatial and temporal expression of genes, as well as intensity of the cellular response. Indeed, in the adult body, angiogenesis is constantly suppressed; the levels of anti-angiogenic molecules predominate in every tissue. However, failure of the regulatory processes that inhibit angiogenesis leads to the excessive generation of blood vessels that participate in cancer progression [30]. Higher VEGF expression can increase tumor-related angiogenesis and metastasis [31]. The role of angiogenesis as a prognostic factor of carcinogenesis and cancer progression, however, is still controversial [32, 33]. Weidner et al. [3] first reported a direct correlation between the incidence of metastasis and the number and density of blood vessels in invasive breast carcinomas. Similar studies have made this association in gastrointestinal [34] and colorectal cancers [32, 35–39]. An association between elevated angiogenesis and both a high prevalence of metastases and a subsequent decrease in survival has been reported for a vast majority of solid tumors [32, 35–39]. Several studies have revealed high angiogenic activity in CRC, which was more likely correlated with aggressive histological and pathological characteristics including parietal invasion, tumor stage, grade of tumor differentiation, metastatic rates, and poor survival rates [32, 40, 41]. Also, Gurzu et al. [32] reported that augmented levels angiogenesis in CRC were higher during early stages of tumor proliferation, but did not progressively increase as the tumors advanced. For these reasons, anti-angiogenesis is one possible target for cancer prevention and therapy.

However, anti-angiogenesis can induce the metastatic potential of cancer. Inhibition of VEGF/VEGF receptor (VEGFR) signaling causes a decrease in nutrient and oxygen levels, inducing hypoxia [42]. One of the crucial steps in the cellular response to hypoxia is the stabilization of hypoxia-inducible factor (HIF)-1 in low-oxygen conditions. As a consequence, genes directly regulated by this transcription factor are activated. Because HIF-1 modulates the transcription of genes involved in glycolytic metabolism, oxygen consumption, survival, angiogenesis, migration, and invasion, its stabilization has a dramatic impact on the gene expression profile and ultimately on the behavior of the cells [43]. For example, cancer cells react to the hypoxia caused by anti-angiogenic signals by reprogramming their metabolism, thus increasing the uptake of glucose to sustain energy production through glycolysis. This phenomena is referred to as the Warburg effect [43]. Also, the epithelial-mesenchymal transition when stabilized HIF-1 transactivates the *Met* proto-oncogene, the receptor for hepatocyte growth factor [44]. In addition, recent data suggest that VEGF supplementation may inhibit invasion and epithelial-mesenchymal transition of cancer cells [45]. Therefore, for cancer prevention and therapy, it may be important to keep VEGF expression levels within a normal range. Previous reports showed that polymorphic events affecting the mRNA expression of *VEGF* and *VEGFR* genes could contribute to the survival duration of cancer patients receiving anti-angiogenic treatment [46–49]. The *VEGF* -2578A/-1498C/-634G haplotype showed a shorter survival time in multiple myeloma patients treated with thalidomide [46]. The progression-free survival duration of metastatic renal cell carcinoma patients who received bevacizumab treatment varied between *VEGFR1* rs7993418 alleles [47]. *VEGF* -2578CC, -1498TT, and -634CC were associated with a poorer prognosis and progression-free survival duration in advanced renal cell carcinoma patients receiving first-line sunitinib [48]. *VEGF* -634CC displayed relatively shorter progression free survival duration in advanced castration-resistant prostate cancer patients treated with metronomic cyclophosphamide [49].

We identified an association between lower mRNA expression and RC risk in the *VEGF* 1451T carrier. Moreover, *VEGF* 1451T carrier combined with MetS was linked to increased RC risk, whereas the combination of higher mRNA expression in the *VEGF* 1725A carrier and MetS was linked to elevated CC risk. *VEGF* 1451T carrier could directly contribute to RC risk, but the *VEGF* 1725A allele could not have influence on CC risk alone. In other studies, weakened VEGF/VEGFR signaling was shown to cause a decrease in oxygen levels, and the HIF-1 transcription factor is stabilized in insulin resistance conditions [42, 50]. We hypothesize that low oxygen conditions in *VEGF* 1451T carrier and HIF-1 activation in MetS patients may lead to early cancer development. Activated HIF-1 drives the transcription of over 60 genes that are involved in cancer biology, including angiogenesis, cell survival, and glucose metabolism [51]. *VEGF* 1725A carrier may influence a pathological angiogenesis step after persistent HIF-1 activation.

Genetic variation in the 3′-UTR region could affect the stability and translation of the mRNA through altered miRNA binding affinity. Currently, there are no data to directly show altered miRNA binding activity depending on *VEGF* 3′-UTR polymorphisms. Further studies are needed to directly test for miRNA binding activity to *VEGF* 3′-UTR polymorphisms to determine the mechanism by which these polymorphisms may influence cellular proliferation and cancer progression. These studies may have great clinical impact for all diseases related to abnormal angiogenesis and hypoxic conditions.

There are several limitations in this study. First, the mechanism by which 1451C > T, 1612G > A, and 1725G > A polymorphisms in the *VEGF* gene affect development of CRC is still unclear. Further studies of whole *VEGF* sequence variants and their biological functions would uncover the role of these *VEGF* polymorphisms and haplotypes in the development and progression of CRC. Second, the present study lacked information regarding additional environmental risk factors (smoking, alcohol intake, caffeine intake, red meat intake, and multivitamin use) and clinical characteristics (survival time, relapse, death, chemotherapy, and radiotherapy) in the CRC patient cohort. These factors may contribute to overall CRC risk. Lastly, the population of this study was restricted to patients of Korean ethnicity. Because frequencies of genetic polymorphisms often vary between ethnic groups, more studies in diverse ethnic populations are warranted to clarify the association between *VEGF* 3′-UTR polymorphisms and CRC.

## Conclusion

We investigated the involvement of *VEGF* polymorphisms 1451C > T, 1612G > A, and 1725G > A with CRC susceptibility in the present study. *VEGF* 1451C > T and 1725G > A could contribute to CRC susceptibility when combined with the presence of MetS. Moreover, VEGF mRNA expression varied in tumor tissues depending on the combination of 3′-UTR polymorphic alleles present. Although results from our study provide the first evidence for *VEGF* 1451C > T, 1612G > A, and 1725G > A as potential biomarkers for CRC prevention, a prospective study on a larger cohort of patients is warranted to validate these findings.

## Electronic supplementary material

Additional file 1: Table S1: The frequencies of MetS and *VEGF* 3′-UTR genotypes according to clinicopathological features of CRC. (DOC 54 KB)

## Abbreviations

(AJCC): American Joint Committee on Cancer
(AOR): Adjusted odds ratios
(BMI): Body mass index
(BP): Blood pressure
(CC): Colon cancer
(CI): Confidence interval
(CRC): Colorectal cancer
(DM): Diabetes mellitus
(TG): Triglycerides
(FBS): Fasting blood sugar
(HDL-C): High density lipoprotein-cholesterol
(HIF): Hypoxia-inducible factor
(HTN): Hypertension
(HWE): Hardy-Weinberg equilibrium
(MDR): Multifactor dimensionality reduction
(MetS): Metabolic syndrome
(OR): Odds ratio
(PCR): Polymerase chain reaction
(qRT-PCR): Quantitative real-time PCR
(RC): Rectal cancer
(RR): Relative risk
(SD): Standard deviation
(SE): Standard error
(TNM): Tumor node metastasis
(VEGF): Vascular endothelial growth factor
(VEGFR): VEGF receptor.

## References

[1] Jemal A, Siegel R, Ward E, Hao Y, Xu J, Thun MJ (2009). Cancer statistics, 2009. CA Cancer J Clin.

[2] Figueredo A, Coombes ME, Mukherjee S (2008). Adjuvant therapy for completely resected stage II colon cancer. Cochrane Database Syst Rev.

[3] Weidner N, Semple JP, Welch WR, Folkman J (1991). Tumor angiogenesis and metastasis–correlation in invasive breast carcinoma. N Engl J Med.

[4] Dvorak HF, Brown LF, Detmar M, Dvorak AM (1995). Vascular permeability factor/vascular endothelial growth factor, microvascular hyperpermeability, and angiogenesis. Am J Pathol.

[5] Yancopoulos GD, Davis S, Gale NW, Rudge JS, Wiegand SJ, Holash J (2000). Vascular-specific growth factors and blood vessel formation. Nature.

[6] Hansen TF, Jakobsen A (2011). Clinical implications of genetic variations in the VEGF system in relation to colorectal cancer. Pharmacogenomics.

[7] Wang L, Liu W, Jiang W, Lin J, Jiang Y, Li B, Pang D (2012). A miRNA binding site single-nucleotide polymorphism in the 3′-UTR region of the IL23R gene is associated with breast cancer. PLoS One.

[8] Sebio A, Paré L, Páez D, Salazar J, González A, Sala N, del Río E, Martín-Richard M, Tobeña M, Barnadas A, Baiget M (2013). The LCS6 polymorphism in the binding site of let-7 microRNA to the KRAS 3′-untranslated region: its role in the efficacy of anti-EGFR-based therapy in metastatic colorectal cancer patients. Pharmacogenet Genomics.

[9] Yang P, Tang R, Zhu J, Zou L, Wu R, Zhou H, Mao Y, Li R, Hua D, Wang W, Zhang H (2013). A functional variant at miR-24 binding site in B7-H2 alters susceptibility to gastric cancer in a Chinese Han population. Mol Immunol.

[10] Zhou L, Zhang X, Li Z, Zhou C, Li M, Tang X, Lu C, Li H, Yuan Q, Yang M (2013). Association of a genetic variation in a miR-191 binding site in MDM4 with risk of esophageal squamous cell carcinoma. PLoS One.

[11] Gami AS, Witt BJ, Howard DE, Erwin PJ, Gami LA, Somers VK, Montori VM (2007). Metabolic syndrome and risk of incident cardiovascular events and death: a systematic review and meta-analysis of longitudinal studies. J Am Coll Cardiol.

[12] Giovannucci E (2007). Metabolic syndrome, hyperinsulinemia, and colon cancer: a review. Am J Clin Nutr.

[13] Pais R, Silaghi H, Silaghi AC, Rusu ML, Dumitrascu DL (2009). Metabolic syndrome and risk of subsequent colorectal cancer. World J Gastroenterol.

[14] Ahmed RL, Schmitz KH, Anderson KE, Rosamond WD, Folsom AR (2006). The metabolic syndrome and risk of incident colorectal cancer. Cancer.

[15] Colangelo LA, Gapstur SM, Gann PH, Dyer AR, Liu K (2002). Colorectal cancer mortality and factors related to the insulin resistance syndrome. Cancer Epidemiol Biomarkers Prev.

[16] La Vecchia C, D’Avanzo B, Negri E, Franceschi S (1991). History of selected diseases and the risk of colorectal cancer. Eur J Cancer.

[17] Le Marchand L, Wilkens LR, Kolonel LN, Hankin JH, Lyu LC (1997). Associations of sedentary lifestyle, obesity, smoking, alcohol use, and diabetes with the risk of colorectal cancer. Cancer Res.

[18] Kono S, Honjo S, Todoroki I, Nishiwaki M, Hamada H, Nishikawa H, Koga H, Ogawa S, Nakagawa K (1998). Glucose intolerance and adenomas of the sigmoid colon in Japanese men (Japan). Cancer Causes Control.

[19] Will JC, Galuska DA, Vinicor F, Calle EE (1998). Colorectal cancer: another complication of diabetes mellitus?. Am J Epidemiol.

[20] Larsson SC, Orsini N, Wolk A (2005). Diabetes mellitus and risk of colorectal cancer: a meta-analysis. J Natl Cancer Inst.

[21] Trevisan M, Liu J, Muti P, Misciagna G, Menotti A, Fucci F, Risk Factors and Life Expectancy Research Group (2001). Markers of insulin resistance and colorectal cancer mortality. Cancer Epidemiol Biomarkers Prev.

[22] Dong J, Dai J, Shu Y, Pan S, Xu L, Chen W, Wang Y, Jin G, Ma H, Zhang M, Hu Z, Shen H (2010). Polymorphisms in EGFR and VEGF contribute to non-small-cell lung cancer survival in a Chinese population. Carcinogenesis.

[23] Tahara T, Shibata T, Nakamura M, Yamashita H, Yoshioka D, Hirata I, Arisawa T (2009). Effect of polymorphisms in the 3′ untranslated region (3′-UTR) of vascular endothelial growth factor gene on gastric cancer and peptic ulcer diseases in Japan. Mol Carcinog.

[24] Jang MJ, Jeon YJ, Kim JW, Cho YK, Lee SK, Hwang SG, Oh D, Kim NK (2013). Association of VEGF and KDR single nucleotide polymorphisms with colorectal cancer susceptibility in Koreans. Mol Carcinog.

[25] Shahbazi M, Fryer AA, Pravica V, Brogan IJ, Ramsay HM, Hutchinson IV, Harden PN (2002). Vascular endothelial growth factor gene polymorphisms are associated with acute renal allograft rejection. J Am Soc Nephrol.

[26] Hansen TF, Spindler KL, Lorentzen KA, Olsen DA, Andersen RF, Lindebjerg J, Brandslund I, Jakobsen A (2010). The importance of -460 C/T and +405 G/C single nucleotide polymorphisms to the function of vascular endothelial growth factor A in colorectal cancer. J Cancer Res Clin Oncol.

[27] Krippl P, Langsenlehner U, Renner W, Yazdani-Biuki B, Wolf G, Wascher TC, Paulweber B, Haas J, Samonigg H (2003). A common 936 C/T gene polymorphism of vascular endothelial growth factor is associated with decreased breast cancer risk. Int J Cancer.

[28] Antonacopoulou AG, Kottorou AE, Dimitrakopoulos FI, Triantafyllia V, Marousi S, Koutras A, Kalofonos HP (2011). VEGF polymorphisms may be associated with susceptibility to colorectal cancer: a case–control study. Cancer Biomark.

[29] Maltese P, Canestrari E, Ruzzo A, Graziano F, Falcone A, Loupakis F, Tonini G, Santini D, Magnani M (2009). VEGF gene polymorphisms and susceptibility to colorectal cancer disease in Italian population. Int J Colorectal Dis.

[30] Hanahan D, Folkman J (1996). Patterns and emerging mechanisms of the angiogenic switch during tumorigenesis. Cell.

[31] Crawford Y, Ferrara N (2009). VEGF inhibition: insights from preclinical and clinical studies. Cell Tissue Res.

[32] Gurzu S, Jung J, Azamfirei L, Mezei T, Cîmpean AM, Szentirmay Z (2008). The angiogenesis in colorectal carcinomas with and without lymph node metastases. Rom J Morphol Embryol.

[33] Rodrigo JP, Cabanillas R, Chiara MD, García Pedrero J, Astudillo A, Suárez Nieto C (2009). [Prognostic significance of angiogenesis in surgically treated supraglottic squamous cell carcinomas of the larynx]. Acta Otorrinolaringol Esp.

[34] Kitadai Y (2010). Angiogenesis and lymphangiogenesis of gastric cancer. J Oncol.

[35] Tanigawa N, Amaya H, Matsumura M, Lu C, Kitaoka A, Matsuyama K, Muraoka R (1997). Tumor angiogenesis and mode of metastasis in patients with colorectal cancer. Cancer Res.

[36] Barozzi C, Ravaioli M, D’Errico A, Grazi GL, Poggioli G, Cavrini G, Mazziotti A, Grigioni WF (2002). Relevance of biologic markers in colorectal carcinoma: a comparative study of a broad panel. Cancer.

[37] Saad RS, Liu YL, Nathan G, Celebrezze J, Medich D, Silverman JF (2004). Endoglin (CD105) and vascular endothelial growth factor as prognostic markers in colorectal cancer. Mod Pathol.

[38] De Vita F, Orditura M, Lieto E, Infusino S, Morgillo F, Martinelli E, Castellano P, Romano C, Ciardiello F, Catalano G, Pignatelli C, Galizia G (2004). Elevated perioperative serum vascular endothelial growth factor levels in patients with colon carcinoma. Cancer.

[39] Des Guetz G, Uzzan B, Nicolas P, Cucherat M, Morere JF, Benamouzig R, Breau JL, Perret GY (2006). Microvessel density and VEGF expression are prognostic factors in colorectal cancer. Meta-analysis of the literature. Br J Cancer.

[40] Giatromanolaki A, Stathopoulos GP, Tsiompanou E, Papadimitriou C, Georgoulias V, Gatter KC, Harris AL, Koukourakis MI (1999). Combined role of tumor angiogenesis, bcl-2, and p53 expression in the prognosis of patients with colorectal carcinoma. Cancer.

[41] Svagzdys S, Lesauskaite V, Pavalkis D, Nedzelskiene I, Pranys D, Tamelis A (2009). Microvessel density as new prognostic marker after radiotherapy in rectal cancer. BMC Cancer.

[42] Heath VL, Bicknell R (2009). Anticancer strategies involving the vasculature. Nat Rev Clin Oncol.

[43] Semenza GL (2007). Hypoxia and cancer. Cancer Metastasis Rev.

[44] Svagzdys S, Lesauskaite V, Pavalkis D, Nedzelskiene I, Pranys D, Tamelis A (2003). Hypoxia promotes invasive growth by transcriptional activation of the met protooncogene. Cancer Cell.

[45] Lu KV, Chang JP, Parachoniak CA, Pandika MM, Aghi MK, Meyronet D, Isachenko N, Fouse SD, Phillips JJ, Cheresh DA, Park M, Bergers G (2012). VEGF inhibits tumor cell invasion and mesenchymal transition through a MET/VEGFR2 complex. Cancer Cell.

[46] Andersen NF, Vogel U, Klausen TW, Gimsing P, Gregersen H, Abildgaard N, Vangsted AJ (2012). Vascular endothelial growth factor (VEGF) gene polymorphisms may influence the efficacy of thalidomide in multiple myeloma. Int J Cancer.

[47] Lambrechts D, Claes B, Delmar P, Reumers J, Mazzone M, Yesilyurt BT, Devlieger R, Verslype C, Tejpar S, Wildiers H, de Haas S, Carmeliet P, Scherer SJ, Van Cutsem E (2012). VEGF pathway genetic variants as biomarkers of treatment outcome with bevacizumab: an analysis of data from the AViTA and AVOREN randomised trials. Lancet Oncol.

[48] Scartozzi M, Bianconi M, Faloppi L, Loretelli C, Bittoni A, Del Prete M, Giampieri R, Maccaroni E, Nicoletti S, Burattini L, Minardi D, Muzzonigro G, Montironi R, Cascinu S (2013). VEGF and VEGFR polymorphisms affect clinical outcome in advanced renal cell carcinoma patients receiving first-line sunitinib. Br J Cancer.

[49] Orlandi P, Fontana A, Fioravanti A, Di Desidero T, Galli L, Derosa L, Canu B, Marconcini R, Biasco E, Solini A, Francia G, Danesi R, Falcone A, Bocci G (2013). VEGF-A polymorphisms predict progression-free survival among advanced castration-resistant prostate cancer patients treated with metronomic cyclophosphamide. Br J Cancer.

[50] Girgis CM, Cheng K, Scott CH, Gunton JE (2012). Novel links between HIFs, type 2 diabetes, and metabolic syndrome. Trends Endocrinol Metab.

[51] Paul SA, Simons JW, Mabjeesh NJ (2004). HIF at the crossroads between ischemia and carcinogenesis. J Cell Physiol.

[CR52] The pre-publication history for this paper can be accessed here: http://www.biomedcentral.com/1471-2407/14/881/prepub

