# Supplementary material for: Interplay between 3′-UTR polymorphisms in the vascular endothelial growth factor (VEGF) gene and metabolic syndrome in determining the risk of colorectal cancer in Koreans
Source: BMC Cancer. 2014 Nov 25;14:881. doi: 10.1186/1471-2407-14-881 (PMC4289193; doi:10.1186/1471-2407-14-881)
Supplement: Supplementary file 1 — Additional file 1: Table S1: The frequencies of MetS and VEGF 3′-UTR genotypes according to clinicopathological features of CRC. (DOC 54 KB) [file 12885_2014_5128_MOESM1_ESM.doc]

Additional File 1

| **Table S1** The frequencies of MetS and *VEGF* 3'-UTR genotypes according to clinicopathological features of CRC | | | | | | | | | |
| --- | --- | --- | --- | --- | --- | --- | --- | --- | --- |
| Characteristics | CC | RC | *p* | Tumor<5 cm | Tumor≥5 cm | *p* | TNM stage I+II | TNM stage III+IV | *p* |
| N | 264 | 186 |  | 181 | 269 |  | 231 | 219 |  |
| Without MetS | 166 (62.9) | 113 (60.8) | 0.720 | 108 (59.7) | 171 (63.6) | 0.461 | 142 (61.5) | 137 (62.6) | 0.889 |
| With MetS | 98 (37.1) | 73 (39.2) |  | 73 (40.3) | 98 (36.4) |  | 89 (38.5) | 82 (37.4) |  |
| *VEGF* 1451CC | 180 (68.2) | 109 (58.6) | 0.081 | 120 (66.3) | 169 (62.8) | 0.628 | 148 (64.1) | 141 (64.4) | 0.988 |
| *VEGF* 1451CT | 73 (27.7) | 70 (37.6) |  | 53 (29.3) | 90 (33.5) |  | 74 (32.0) | 69 (31.5) |  |
| *VEGF* 1451TT | 11 (4.2) | 7 (3.8) |  | 8 (4.4) | 10 (3.7) |  | 9 (3.9) | 9 (4.1) |  |
| *VEGF* 1612GG | 195 (73.9) | 141 (75.8) | 0.346 | 141 (77.9) | 195 (72.5) | 0.261 | 172 (74.5) | 164 (74.9) | 0.857 |
| *VEGF* 1612GA | 65 (24.6) | 39 (21.0) |  | 35 (19.3) | 69 (25.7) |  | 53 (22.9) | 51 (23.3) |  |
| *VEGF* 1612AA | 4 (1.5) | 6 (3.2) |  | 5 (2.8) | 5 (1.9) |  | 6 (2.6) | 4 (1.8) |  |
| *VEGF* 1725GG | 226 (85.6) | 157 (84.4) | 0.828 | 157 (86.7) | 226 (84.0) | 0.508 | 199 (86.1) | 184 (84.0) | 0.616 |
| *VEGF* 1725GA | 38 (14.4) | 29 (15.6) |  | 24 (13.3) | 43 (16.0) |  | 32 (13.9) | 35 (16.0) |  |
| *VEGF* 1725AA | 0 (0.0) | 0 (0.0) |  | 0 (0.0) | 0 (0.0) |  | 0 (0.0) | 0 (0.0) |  |
| *VEGF* 1451C/1612G/1725G | 355 (67.2) | 236 (63.4) | 0.247 | 245 (67.7) | 346 (64.3) | 0.774 | 303 (65.6) | 288 (65.8) | 0.854 |
| *VEGF* 1451T/1612G/1725G | 95 (18.0) | 84 (22.6) |  | 69 (19.1) | 110 (20.4) |  | 92 (19.9) | 87 (19.9) |  |
| *VEGF* 1451C/1612A/1725G | 40 (7.6) | 23 (6.2) |  | 24 (6.6) | 39 (7.2) |  | 35 (7.6) | 28 (6.4) |  |
| *VEGF* 1451C/1612A/1725A | 33 (6.3) | 28 (7.5) |  | 21 (5.8) | 40 (7.4) |  | 30 (6.5) | 31 (7.1) |  |
| *VEGF* 1451C/1612G/1725A | 5 (0.9) | 1 (0.3) |  | 3 (0.8) | 3 (0.6) |  | 2 (0.4) | 4 (0.9) |  |
| Metabolic syndrome (MetS), Vascular endothelial growth factor (VEGF), Colorectal cancer (CRC), Colon cancer (CC), Rectal cancer (RC), Tumor node metastasis (TNM). *p* values were calculated by chi-square test. | | | | | | | | | |
